# Retraction Note

**Published:** 2018-05-01

For a mistake made in the *IJOTM* Editorial Office, one of the articles that had already been published in October 2017 issue of the Journal,^1^ has inadvertently republished in the January 2018 issue. Herein, for ethical concern about duplicate publication, we retract the recent article appeared in the January issue (PMID 29531646). We apologize for any inconvenience this may cause.

.

*IJOTM*
